# Carbapenem Resistance in *Acinetobacter calcoaceticus-baumannii* Complex Isolates From Kathmandu Model Hospital, Nepal, Is Attributed to the Presence of *bla*_OXA-23-like_ and *bla*_NDM-1_ Genes

**DOI:** 10.1155/2024/8842625

**Published:** 2024-08-12

**Authors:** Anupama Gurung, Rajindra Napit, Basudha Shrestha, Binod Lekhak

**Affiliations:** ^1^ Central Department of Microbiology Tribhuvan University, Kirtipur, Nepal; ^2^ Central Department of Biotechnology Tribhuvan University, Kirtipur, Nepal; ^3^ Department of Microbiology Kathmandu Model Hospital, Putalisadak, Kathmandu, Nepal

**Keywords:** ACB complex, *bla*_NDM-1_ gene, *bla*_OXA-23-like_ gene, *bla*_OXA-24-like_ gene, carbapenem resistance

## Abstract

The *Acinetobacter calcoaceticus-baumannii* (ACB) complex, also known as ACB complex, consists of four bacterial species that can cause opportunistic infections in humans, especially in hospital settings. Conventional therapies for susceptible strains of the ACB complex include broad-spectrum cephalosporins, *β*-lactam/*β*-lactamase inhibitors, and carbapenems. Unfortunately, the effectiveness of these antibiotics has declined due to increasing rates of resistance. The predominant resistance mechanisms identified in the ACB complex involve carbapenem-resistant (CR) oxacillinases and metallo-*β*-lactamases (MBLs). This research, conducted at Kathmandu Model Hospital in Nepal, sought to identify genes associated with CR, specifically *bla*NDM-1, *bla*OXA-23-like, and *bla*OXA-24-like genes in carbapenem-resistant *Acinetobacter calcoaceticus-baumannii* (CR-ACB) complex. Additionally, the study is aimed at identifying the ACB complex through the sequencing of the 16s rRNA gene. Among the 992 samples collected from hospitalized patients, 43 (approximately 4.334%) tested positive for the ACB complex. These positive samples were mainly obtained from different hospital units, including intensive care units (ICUs); cabins; and neonatal, general, and maternity wards. The prevalence of infection was higher among males (58.14%) than females (41.86%), with the 40–50 age group showing the highest infection rate. In susceptibility testing, colistin and polymyxin B exhibited a susceptibility rate of 100%, whereas all samples showed resistance to third-generation cephalosporins. After polymyxins, gentamicin (30.23%) and amikacin (34.88%) demonstrated the highest susceptibility. A substantial majority (81.45%) of ACB complex isolates displayed resistance to carbapenems, with respiratory and pus specimens being the primary sources. Polymerase chain reaction (PCR) revealed that the primary CR gene within the ACB complex at this hospital was *bla*_OXA-23-like_, followed by *bla*_NDM-1_. To ensure the accuracy of the phenotypic assessment, 12 samples were chosen for 16s rRNA sequencing using Illumina MiSeq™ to confirm that they are *Acinetobacter* species. QIIME 2.0 analysis confirmed all 12 isolates to be *Acinetobacter* species. In the hospital setting, a substantial portion of the ACB complex carries CR genes, rendering carbapenem ineffective for treatment.

## 1. Background

The genus *Acinetobacter* encompasses a diverse and heterogeneous collection of bacteria, many of which have the potential to induce different forms of opportunistic infections in humans [1]. Nevertheless, the prevalent *Acinetobacter* species found in hospital environments belong to the *Acinetobacter calcoaceticus-baumannii* (ACB) complex [2]. The ACB complex poses a concerning nosocomial threat, primarily linked to epidemic outbreaks of infections that are seldom encountered outside clinical environments. This group includes clinically derived species such as *Acinetobacter baumannii*, *Acinetobacter nosocomialis* (previously genospecies 13TU), and *Acinetobacter pittii* (previously genospecies 3), along with environmentally sourced *Acinetobacter calcoaceticus* within the genus [3]. They are commonly known as the ACB complex, given their close genetic ties, which pose challenges for differentiation using conventional clinical microbiology laboratory methods relying on observable traits [4]. Among these species, *Acinetobacter baumannii* stands out as the most concerning pathogen for healthcare institutions and has emerged as its most notable representative [5].

The recommended treatment guidelines for antibiotic-susceptible ACB complex include broad-spectrum cephalosporin (such as ceftazidime or cefepime), *β*-lactam/*β*-lactamase inhibitors (including sulbactam), and carbapenem (such as imipenem, meropenem, or doripenem) [6]. To mitigate the risk of resistance development, these antibiotics are occasionally combined with an aminoglycoside or an antipseudomonal fluoroquinolone. In cases of multidrug-resistant (MDR) and carbapenem-resistant *Acinetobacter calcoaceticus-baumannii* (CR-ACB) complex, polymyxins (polymyxin B and colistin) and tetracyclines (minocycline and tigecycline) are the preferred drugs [7]. Carbapenem, belonging to the broadest spectrum *β*-lactam class of antibiotics, exhibits bactericidal activity against a wide range of gram-positive and gram-negative aerobes, as well as anaerobes. Carbapenems have been the primary antimicrobial agent against *Acinetobacter baumannii* infections since 1990 [8]. However, due to increasing rates of CR, their efficacy has significantly declined [5].

Historically, Ambler's molecular structure classification [9] and Bush-Jacoby-Medeiros' functional classification [10, 11] have served as frameworks for categorizing *β*-lactamases. Ambler's classification divides these enzymes into four groups, A, B, C, and D, based on distinctive motifs formed by the fundamental sequences constituting the protein molecules. Class B enzymes require zinc as a metal cofactor for their activity, whereas class A, C, and D enzymes utilize a serine residue as their active core [12]. In the ACB complex, all four classes of *β*-lactamases are present, with carbapenemases belonging to classes A, B, and C, while cephalosporinases are categorized as class C enzymes. In the ACB complex, the primary mechanism of CR involves enzymatic breakdown, leading to the inactivation of carbapenems. This process is chiefly orchestrated by carbapenemase enzymes [13].

Oxacillinases form a significant category of carbapenemases within the ACB complex [14]. There are four primary groups of CR oxacillinases: OXA-23-like, OXA-24/40-like, OXA-58-like, and the intrinsic OXA-51-like genes [15]. Among these genes, OXA-51-like, OXA-23-like, and OXA-58-like are influenced by insertion sequences that provide promoter sequences facilitating their gene expression. The associated insertion sequences include *ISAba1*, *ISAba2*, *ISAba3*, and *ISAba4* [16]. Metallo-*β*-lactamases (MBLs), or class B *β*-lactamases, represent the second most common method of CR in the ACB complex. Despite being less prevalent carbapenemases in the ACB complex compared to oxacillinases, MBLs possess more potent carbapenem-hydrolyzing properties (100–1000-fold) [5, 17]. Lastly, Ambler class A carbapenemase is a rare phenomenon within the ACB complex [18].

The exploration of antimicrobial resistance (AMR) genes at the genomic level is crucial for understanding resistance mechanisms. However, there has been a lack of molecular investigations into AMR in the ACB complex in Nepal [19, 20]. Up to the time of this research, only three molecular studies had been undertaken to identify the presence of AMR genes in the ACB complex in Nepal [21–23]. Previous research in Kathmandu Model Hospital focused mainly on phenotypic assessments of AMR [17, 24–28]. Specifically, within the timeframe of this study at Kathmandu Model Hospital, there had been no instances of previous molecular investigations to comprehend the presence of AMR genes within the ACB complex [29, 30]. Since knowledge about AMR genes within the ACB complex primarily relied on just a few studies [21–23], this research is aimed at determining whether the AMR genes, as identified in earlier studies, were apparent in ACB isolates obtained from Kathmandu Model Hospital, Nepal.

Based on previous investigations in another hospital [21] and a tertiary care hospital [23] of Nepal, we hypothesized that the *bla*_NDM-1_ and *bla*_OXA-23-like_ carbapenemase genes are among the most widespread carbapenemase genes circulating within the ACB complex in Nepal. Additionally, the *bla*_OXA-24-like_ gene, belonging to the category of commonly encountered carbapenemase genes, had not been identified in any research conducted in Nepal at the time of this study. However, given its previous isolation in neighboring countries like India and China, there was conjecture regarding its potential presence [31, 32]. Therefore, this study specifically focuses on the prevalence of *bla*_NDM-1_, *bla*_OXA-24-like_, and *bla*_OXA-23-like_ carbapenemase genes in CR-ACB complex isolates obtained from inpatients at Kathmandu Model Hospital.

## 2. Material and Methods

### 2.1. Study Design and Selection of Participants

A prospective cross-sectional study was conducted to assess the prevalence of CR-ACB complex among inpatients (hospital-admitted individuals) and to identify the presence of *bla*_NDM-1_, *bla*_OXA-23-like_, and *bla*_OXA-24-like_ genes associated with CR. The sample collection took place between June 2018 and November 2018 at Kathmandu Model Hospital. A total of 992 clinical samples from inpatients at Kathmandu Model Hospital during that period were chosen as the study cohort, irrespective of age, gender, or clinical symptoms. The variables considered in this study included age, sex, specimen types, antibiotic susceptibility profiles, CR, carbapenemase production, and polymerase chain reaction (PCR) detection of *bla*_NDM-1_, *bla*_OXA-23-like_, and *bla*_OXA-24-like_ genes. The samples utilized in this investigation originated from diverse sources, including tracheal aspirate, sputum, blood, pus, wound swab, tissue, urine, bed sore, and various types of device tips such as endotracheal (ET) tip, central venous catheter (CVC) tip, and urinary catheter tip (CT). Moreover, body fluids such as cerebrospinal fluid (CSF), pleural fluid, peritoneal fluid, pericardial fluid, synovial fluid, pus drain, and bile were also included. Ethical approval for the study was obtained from the Institutional Review Board (IRC) of Phect Nepal in June 2018 (ML/04 - 2018), as well as the Nepal Health Research Council (NHRC).

### 2.2. Bacteria Culture, Isolation, and Initial Identification

Nine hundred ninety-two collected specimens were streaked on MacConkey agar, blood agar, and chocolate agar (HiMedia, India) and incubated at 37°C in aerobic condition for 24 h. The ACB complex was identified using criteria such as colony appearance, gram staining, the catalase test, and biochemical examinations. Following overnight incubation, distinct colonies exhibiting late lactose fermentation characteristics of the ACB complex were observed on MacConkey agar. These colonies underwent further assessment. Gram-negative coccobacilli with a positive catalase test, a negative oxidase test, lacking motility, testing negative for indole production, demonstrating citrate utilization, displaying variable urease activity, and showing no production of hydrogen sulfide in the triple sugar iron agar (TSIA) medium were recognized as members of the ACB complex. To ensure the accuracy of the phenotypic assessment, 12 samples were chosen for 16s rRNA sequencing to confirm that they are *Acinetobacter* species. Once the complete identification was achieved, the isolates were preserved on 0.3% agar-agar at room temperature.

### 2.3. Antibiotic Susceptibility Testing (AST)

The ACB complex underwent an AST using a modified Kirby–Bauer disc diffusion technique conducted on Mueller Hinton agar (MHA; HiMedia, India) [33]. Antibiotics used for performing susceptibility testing were ciprofloxacin (5 *μ*g), levofloxacin (5 *μ*g), ceftriaxone (30 *μ*g), cefotaxime (30 *μ*g), cotrimoxazole (1.25/23.75 *μ*g), meropenem (10 *μ*g), imipenem (10 *μ*g), tigecycline (15 *μ*g), gentamicin (10 *μ*g), colistin (10 *μ*g), amikacin (30 *μ*g), chloramphenicol (30 *μ*g), cefepime (30 *μ*g), doxycycline (30 *μ*g), piperacillin/tazobactam (75/30 *μ*g), polymyxin B (300 units), and cefoperazone/sulbactam (100/10 *μ*g) (HiMedia, India). The result of the susceptibility test was interpreted according to the CLSI guidelines 2018 (sensitive, intermediate, and resistant) [34]. While interpreting the results for colistin, polymyxin B, and tigecycline breakpoints, it is worth noting that the guidelines used were those provided by EUCAST for Enterobacteriaceae, not specifically for *Acinetobacter* spp. [35]. ATCC 25922 *Escherichia coli*, ATCC 29213 *Staphylococcus aureus*, and ATCC 27853 *Pseudomonas aeruginosa* were used for quality control tests of antibiotics.

### 2.4. CR and Carbapenemase Production Testing

CR was identified through susceptibility testing with imipenem and meropenem, utilizing the Kirby–Bauer disc diffusion method. Isolates exhibiting an imipenem zone of inhibition ≤ 18 mm and a meropenem zone of inhibition ≤ 14 mm were classified as CR-ACB complex [34]. The assessment of carbapenemase activity at the phenotypic level was carried out using the modified Hodge test (MHT) with *E. coli* ATCC 25922 and a meropenem disc. A positive control for the test involved *bla*_NDM-1_ gene PCR-positive *Klebsiella pneumoniae* [36].

### 2.5. Molecular Detection of Genes

Bacterial DNA extraction was carried out using the cetyltrimethylammonium bromide (CTAB)/NaCl method [37]. In this process, approximately 1–3 mL of overnight Luria Bertani broth containing a few colonies of isolates underwent centrifugation at 12,000 rpm for 10 min. After discarding the supernatant, the pellet was suspended in 567 *μ*L of TE buffer (Tris EDTA, pH 7.4) and subjected to extraction using the CTAB/NaCl method. Subsequently, the DNA was resuspended in 50 *μ*L of TE buffer and stored at −20°C until further use [38].

PCR was performed for the detection of *bla*_NDM-1_, *bla*_OXA-23-like_, *bla*_OXA-24-like_, and 16s rRNA genes. Amplification of carbapenemase genes was carried out using the primer set from Eurofins Genomics, India (Table S1) [3, 39] and 16s rRNA primer (Macrogen, Korea) (Table S1) [40]. The reaction mixture for CR genes, with a total volume of 10 *μ*L, was prepared using 1 *μ*L of template DNA, 1 *μ*L each of forward and reverse primers (10 pmol), 2 *μ*L nuclease-free water (Thermo Fisher Scientific, India), and 5 *μ*L of 2× PCR master mix (QIAGEN, Germany). Amplification was conducted in a Biorad T100 thermal cycler, following the PCR conditions mentioned in Table S2. The resulting substance was observed in a 2% agarose gel, which underwent electrophoresis at 100 volts for 45 min.

### 2.6. 16s rRNA Amplification

For the amplification of the 16s rRNA gene, a reaction volume of 15 *μ*L was employed, comprising 1.5 *μ*L of DNA template, 3 *μ*L each of forward and reverse primers (10 pmol), and 7.5 *μ*L of 2× KAPA HiFi HotStart ReadyMix (KAPA Biosystem, USA). The amplification conditions are provided in Table S2. The resulting product was visualized in a 2% (*w*/*v*) agarose gel, which underwent electrophoresis at 90 volts for 90 min.

A total of 12 isolates, which exhibited a positive result for the *bla*_NDM-1_ gene, underwent 16s rRNA sequencing on the Illumina MiSeq™ system. The amplification of the 16s rRNA gene was carried out using primers targeting the V3 and V4 regions of the prokaryotic 16s ribosomal RNA gene [40]. The quantification of PCR products was performed using the Qubit™ 3 Fluorometer, followed by multiplexing at an even concentration. Subsequently, the samples were subjected to sequencing with a 300 bp (2 × 150 bp) paired-end configuration using the Illumina MiSeq platform (Illumina, Inc., USA) [41].

### 2.7. Library Preparation and Sequencing

For each sample, 1 ng of the amplified PCR products underwent processing with the Illumina MiSeq Nextera XT DNA Library Preparation Kit (Illumina, Inc., USA). The construction of a paired-end library was carried out with an insert size of 500 bp for the 12 isolates positive for *bla*_NDM-1_. Following the purification of PCR products using AMPure XP beads (Agencourt, USA), they underwent tagmentation and indexing using the Nextera XT Index V2 Kit (Illumina, Inc., USA). The cleaned products were quantified and evaluated once more, employing the Qubit Fluorometer (Invitrogen, USA) and the Agilent bioanalyzer with the DNA 1000 kit (Agilent Technologies, UK). Ultimately, all samples were pooled at a concentration of 4 nm and underwent paired-end sequencing with a 300 bp configuration (2 × 151 bp) on the MiSeq platform (Illumina, USA).

### 2.8. Data Analysis

The results obtained were analyzed using the Statistical Package for Social Sciences (SPSS) software (Version 23.0), with the chi-square test employed to determine the *p* value. Gephi 0.9 was utilized as the tool for network analysis.

### 2.9. 16s rRNA Bacterial Taxonomic Profiling

Data were analyzed using the QIIME Version 2.0 pipeline [42]. Raw sequences were demultiplexed and then quality-filtered using demux in QIIME. The Silva_132_release database was used to assign taxonomy [43].

## 3. Results

This research involved analyzing 992 clinical inpatient samples sent to the Department of Microbiology at Kathmandu Model Hospital, Nepal, for aerobic bacterial culture and AST. Among these samples, 43 (4.334%) tested positive for the ACB complex. These positive samples were obtained from hospitalized patients in different hospital wards, such as intensive care units (ICUs), cabin wards, neonatal wards, general wards, and maternity wards.

### 3.1. Distribution of ACB Complex in Different Specimens, Age Categories, and Gender

The highest number of ACB complex isolates was found in cultures of tracheal aspirate and sputum, both accounting for 18.6%. An equal number of isolates were obtained from cultures of ET tubes, blood, and pus, each representing 11.63%. Comparatively fewer ACB complex isolates were recovered from cultures of wound swabs (9.302%), tissue (6.98%), CSF (2.325%), bile (2.325%), CT (2.325%), urine (2.325%), and bed sores (2.325%) (Table 1).

The research indicated a greater proportion of ACB complex isolates in males (60.47%) compared to females (39.53%) (Table 1). ACB complex isolates retrieved from respiratory samples, as well as from blood and tissue, were more prevalent in males when compared to females (Table 1). In a similar vein, patients in the age bracket of 40–50 years exhibited a higher infection rate than individuals in other age groups, while the age groups < 10 and 10–20 had the lowest infection rates.

### 3.2. Antimicrobial Susceptibility Testing

**Figure 1:**
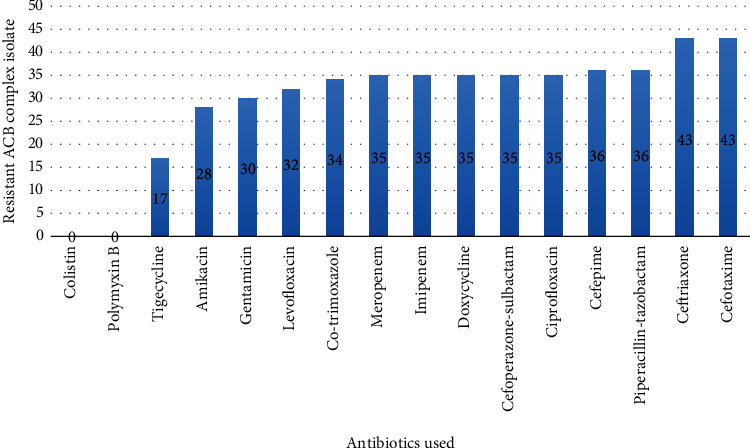
Antibiotic resistance pattern of 43 ACB complex isolates against 16 different antibiotics.

Among the total 43 isolates of the ACB complex, both colistin and polymyxin B demonstrated complete susceptibility, with all 43 isolates (100%) being susceptible. In contrast, all isolates exhibited resistance to third-generation cephalosporins (i.e., ceftriaxone and cefotaxime) (Figure 1). Tigecycline showed a susceptibility rate of 60.47%, while levofloxacin exhibited a susceptibility rate of 25.58%, and cotrimoxazole had a susceptibility rate of 20.93%. A relatively small proportion of ACB complex isolates displayed sensitivity to carbapenems (18.6%). Interestingly, the ACB complex showed a higher sensitivity to gentamicin (30.23%) and amikacin (34.88%) compared to carbapenems. Only a limited number of isolates were susceptible to ciprofloxacin (18.6%), doxycycline (18.6%), cefoperazone/sulbactam (18.6%), piperacillin/tazobactam (16.28%), and cefepime (16.28%) (Table 2).

Out of 43 isolates, 35 (81.45%) were CR and only 8 (18.6%) were carbapenem sensitive.

### 3.3. Temporal Data on the Occurrence of the CR-ACB Complex in Various Specimen Types

**Figure 2:**
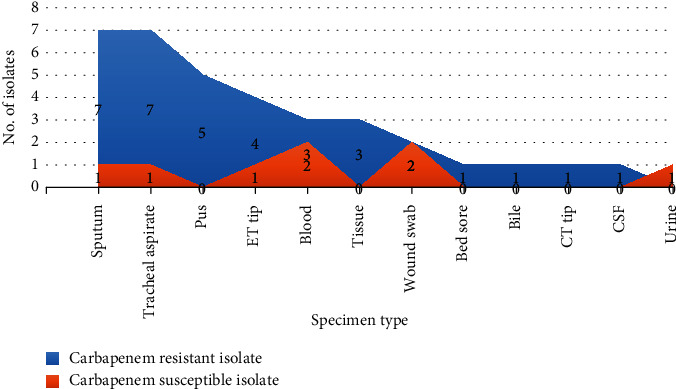
Distribution of carbapenem-resistant and carbapenem-susceptible ACB complex in various specimens.

**Figure 3:**
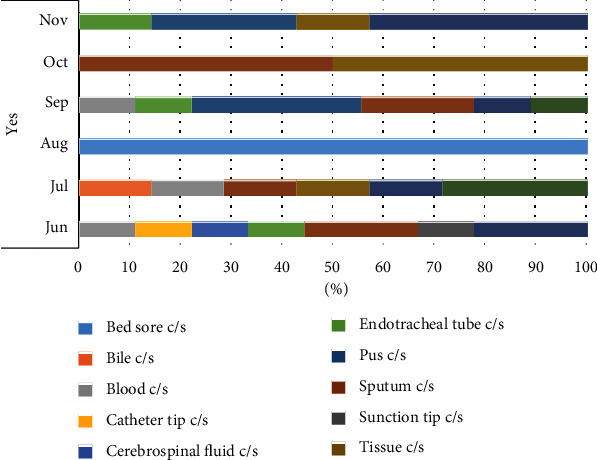
A bar graph illustrating the distribution of ACB complex in various samples throughout the study (June–November 2018).

The predominant source of CR-ACB complex isolates was respiratory specimens, such as sputum, tracheal aspirate, and ET tube tips (Table S3). Respiratory samples accounted for about 54.29% of the identified CR isolates. After respiratory tract samples, pus specimens showed the highest prevalence of CR-ACB complex. No instances of CR-ACB complex were identified in urine samples. A limited number of CR-ACB complex isolates were found in fluid samples, including blood, bile, and CSF (Figure 2). Examining the temporal data (Figure 3), a notable increase in the occurrence of the ACB complex in pus, tissue, and samples from the respiratory system was observed after August. Conversely, the isolation of the ACB complex from wound swabs and blood samples decreased after August. However, before August, the ACB complex was detected in a wider range of specimen types than after that time.

### 3.4. Comparison of AMR Pattern of CR and Carbapenem-Sensitive ACB Complex

In both CR-ACB complex (*n* = 35) and carbapenem-susceptible ACB complex (CS-ACB complex) (*n* = 8), colistin and polymyxin B displayed full susceptibility at 100%, while ceftriaxone and cefotaxime exhibited complete resistance at 100%. Among the CR isolates, the majority demonstrated resistance to cotrimoxazole, piperacillin/tazobactam, ciprofloxacin, and cefepime, constituting 97.1% of the cases. In contrast, the smallest percentage of isolates showed resistance to tigecycline (Table 3).

Among isolates susceptible to carbapenem, most antibiotics displayed complete susceptibility. Out of the 14 antibiotics assessed (excluding meropenem and imipenem), 8 antibiotics exhibited a 100% susceptibility rate, while the remaining 6 demonstrated varying levels of resistance. Among these six antibiotics, ceftriaxone and cefotaxime had the highest resistance rate at 100% (*n* = 8). Following this, doxycycline showed a resistance rate of 37.5% (*n* = 3), while the lowest number of isolates displayed resistance to cefepime, ciprofloxacin, and cefoperazone/sulbactam at 12.5% (*n* = 1), as depicted in (Table 3). It is noteworthy that there was a significant association between susceptibility to carbapenems and susceptibility to other antibiotics (*p* > 0.05), determined through chi-square testing.

### 3.5. Carbapenemase Production, Presence of CR Genes, and 16s rRNA Sequencing Result

The MHT was employed on CR isolates to assess carbapenemase production. Of the 35 CR-ACB complex isolates, 33 (94.28%) showed a positive result in the MHT, characterized by a cloverleaf-shaped indentation. Two samples did not demonstrate clover-leaf indentation which was regarded as MHT negative. Among the 33 carbapenemase-positive CR-ACB complex isolates, 12 (36.36%) were found to carry the *bla*_NDM-1_ gene through PCR analysis (Table 4).

The 16s rRNA gene of the 12 isolates carrying the *bla*_NDM-1_ gene was sequenced using Illumina MiSeq. Analysis via QIIME 2.0 verified that all 12 isolates were *Acinetobacter* species.

Among the 33 carbapenemase-producing CR-ACB complex, 29 (87.87%) tested positive for the *bla*_OXA-23-like_ gene via PCR analysis (Table 5). The two isolates negative in the MHT also lacked the *bla*_OXA-23-like_ e gene. However, neither carbapenemase-producing nor nonproducing isolates showed a positive presence of the *bla*_OXA-24-like_ gene.

### 3.6. Coexistence of *bla*_NDM-1_ and *bla*_OXA-23-like_ Genes

Among the 35 CR-ACB complex isolates, 30 (85.71%) were found to contain CR genes, either individually (*bla*_OXA-23-like_ or *bla*_NDM-1_ gene) or together (both *bla*_OXA-23-like_ and *bla*_NDM-1_ genes) within the same isolates (Tables 4 and 5). The co-occurrence of *bla*_OXA-23-like_ and *bla*_NDM-1_ genes was noted in 11 (35.43%) of these isolates. Among the 12 isolates with the *bla*_NDM-1_ gene, 11 also had the *bla*_OXA-23-like_ gene. Only one isolate had the *bla*_NDM-1_ gene without the *bla*_OXA-23-like_ gene. Furthermore, 18 isolates exclusively carried the *bla*_OXA-23-like_ gene.

### 3.7. Sample-Wise Distribution of *bla*_OXA-23-like_ and *bla*_NDM-1_ Genes

**Figure 4:**
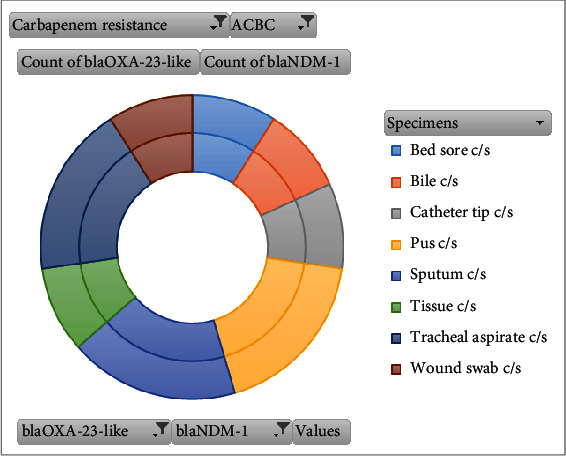
Specimen-wise distribution of *bla*_OXA-23-like_ and *bla*_NDM-1_ genes.

**Figure 5:**
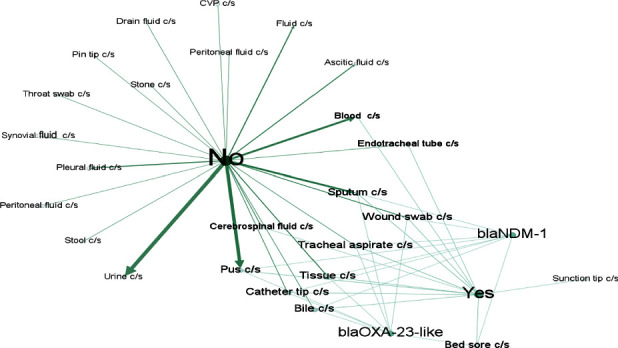
Using Gephi 0.9, a graphical depiction of data related to carbapenem resistance and *bla*_OXA-23-like_ or *bla*_NDM-1_ genes across various samples is presented. “No” indicates sensitivity to carbapenems, while “Yes” denotes resistance. The font size reflects the frequency of variables, with larger fonts indicating higher frequency. The strength of association between variables is represented by the darkness or boldness of connecting arrows.

The examination revealed that *bla*_OXA-23-like_ or *bla*_NDM-1_ genes were predominantly detected in sputum, tracheal aspirate, and pus samples (Figure 4). When visualizing through network analysis, which visualizes relationships between variables, it showed that among CR samples, only one from a suction tip culture lacked any CR genes. Notably, a significant number of samples testing negative for the presence of the ACB complex were obtained from pus, blood, and urine samples (Figure 5).

## 4. Discussion

A lack of knowledge exists regarding the prevalence of genes associated with CR and the current state of the CR-ACB complex in Nepali hospitals [17, 21, 24, 26–28]. The country needs to establish an annual monitoring system to trace the presence of CR-ACB complex genes and their distribution within various healthcare settings [44]. In recognition of this requirement, a research project was carried out at Kathmandu Model Hospital. The aim was to identify three genes (*bla*_NDM-1_, *bla*_OXA-23-like_, and *bla*_OXA-24-like_) from the numerous genes associated with CR within the ACB complex. This investigation represented the inaugural instance of such a study within the ACB complex framework at Kathmandu Model Hospital [29, 30].

The ACB complex stands out as a significant nosocomial pathogen, leading to a variety of diseases. Effectively handling infections caused by the ACB complex poses a challenge due to the tendency of isolated ACB complex strains to demonstrate resistance to multiple drugs [2, 6]. In the absence of proper antimicrobial prescription, these infections can prove to be highly fatal. Carbapenems were traditionally the preferred drugs for treating MDR-ACB complex infections, but recent occurrences of resistance or decreased susceptibility to carbapenems are emerging as a serious clinical issue [8]. In this study, the presence of the ACB complex among hospitalized patients was found to be 4.334%, a rate exceeding the results of prior research conducted in Nepal [25, 45]. Nevertheless, it is lower than the prevalence documented in another study in Nepal [46–48]. The unique pattern observed in our study is likely due to our exclusion criteria (i.e., outpatients). Some studies have included both inpatients and outpatients in their analysis [27, 49]. In this investigation, a higher percentage of isolates were obtained from males (58.14%) compared to females (41.86%), aligning with trends found in previous studies in Nepal (Table 1) [27] and Northern Vietnam [50, 51].

The ACB complex possesses the capability to infect various organs and systems in the human body, allowing its detection in a diverse range of clinical samples [52]. In this study, the ACB complex was identified in various sample types, including bile, CSF, pus, wound swabs, blood, tissue, sputum, tracheal aspirate, urine, bed sores, ET tips, and CT tips (Figures 2 and 3 and Table S3). Despite being present in various samples, a significant majority of ACB complex isolates were found in respiratory samples, such as tracheal aspirate, sputum, and ET tips, constituting 48.83% of the cases. These results are consistent with previously published findings from Nepal [23, 45, 47, 53] and other countries [54, 55]. Respiratory infections are the most common form of *Acinetobacter* infections, occurring predominantly in critically ill patients, especially those admitted to ICUs with mechanical ventilation [56]. Examining the temporal data (Figure 3), it is evident that there was an increase in the incidence of infections from respiratory, pus, and tissue samples starting in August and continuing through November (late summer to early winter months). In another study, a similar trend of elevated respiratory-related infections was noted [57]. Additionally, a study conducted in Western Nepal documented a general increase in *Acinetobacter baumannii* infections during the same month [48].

Regarding susceptibility testing to antimicrobial agents, all isolates of the ACB complex were resistant to third-generation cephalosporins, such as ceftriaxone and cefotaxime (Table 2), which aligns with previous findings [58]. Resistance to fourth-generation cephalosporin (i.e., cefepime) was found to be 83.72%; however, it is noteworthy that a level of resistance (i.e., 65.4% [27], 74.4% [49], 88.6% [59], and 89.79% [48]) has been reported in other studies. The combination of cefoperazone and sulbactam exhibited an even higher susceptibility rate (18.6%) compared to cefepime (Table 2). Sulbactam, serving as a *β*-lactamase inhibitor, along with other inhibitors like clavulanate and tazobactam, possesses intrinsic antibacterial activity against *Acinetobacter* species [60]. Consequently, the cefoperazone/sulbactam combination demonstrated greater antimicrobial activity than other cephalosporins, despite cefoperazone being a third-generation cephalosporin. A similar rationale may be applied to the increased susceptibility of piperacillin/tazobactam. In this study, 65.11% of isolates exhibited resistance to amikacin, and 69.77% were resistant to gentamicin (Table 2). Comparable resistance rates for amikacin (67.24%) and gentamicin (70.68%) were previously documented in Nepal by Bhandari et al. [61].

The resistance rates to imipenem and meropenem were unexpectedly lower at 81.4% in this study, contrary to findings in most other studies conducted within Kathmandu Valley [39, 47, 62] which reported higher resistance rates. However, some studies, like those conducted in Lalitpur District [27], Eastern Nepal [63], and Western Nepal [48], reported lower percentages of resistance. In Nepal, the percentage of CR has significantly increased, with reported resistance rates ranging from 17.24% [61] to 97.7% [23] indicating a critical state of CR. Concerning carbapenemase genes, the most frequently identified one is *bla*_OXA-23-like_ [3, 21]. However, in recent times, *bla*_NDM-1_ has become widespread across various bacteria, primarily carried by *Acinetobacter* spp. and Enterobacteriaceae [39, 64–68]. With this in mind, we conducted PCR testing to identify the presence of *bla*_NDM-1_, *bla*_OXA-24-like_, and *bla*_OXA-23-like_ genes in the CR-ACB complex. Among 35 CR isolates, 82.5% (29/35) tested positive for the *bla*_OXA-23-like_ gene, and 34.28% (12/35) were positive for the *bla*_NDM-1_gene (Tables 4 and 5 and Figures 4 and 5). Among these isolates, 31.42% (11/35) were positive for both *bla*_OXA-23-like_ and *bla*_NDM-1_ genes. The sole isolate contained only the *bla*_NDM-1_gene, and none of the isolates carried the *bla*_OXA-24-like_ gene. In total, 30 isolates (85.71%) contained the tested genes. Notably, five of the isolates did not possess any of the tested genes, which is a study limitation since we did not examine these isolates for other CR genes like *bla*_OXA-58-like_, *bla*_IMP_, *bla*_SIM_, *bla*_VIM_, *bla*_KPC_, *bla*_SME_ [3, 16, 69, 70] or nonenzymatic CR mechanisms such as efflux pumps, loss of porin proteins, and altered penicillin-binding proteins [5]. CR in these cases could potentially be attributed to the mentioned mechanisms. Additionally, two of the isolates tested negative for the MHT, indicating that nonenzymatic methods might be responsible for their CR [71].

In previous molecular investigations carried out in Nepal, a higher incidence of the *bla*_OXA-23-like_ gene was observed compared to the findings of our current research. Two studies demonstrated elevated incidence rates of *the bla*_OXA-23-like_ gene, with 95.08% and 100% [21, 23]. Additional studies conducted between 2012 and 2019 found a gene incidence rate of 36% [68], 63.2% [62], and 69.59% [47]. The prevalence of the *bla*_NDM-1_ gene stood notably higher (34.285%) compared to earlier research findings in Nepal. Three studies reported a prevalence of the *bla*_NDM-1_ gene at 13.6% in Kathmandu, 16.7% in Eastern Nepal [49], and 20.7% in Lalitpur [68], which is significantly lower than the prevalence discovered in our study. This indicates a rising trend and increased dissemination of the *bla*_NDM-1_ gene in Nepal. This trend may be attributed to *Acinetobacter baumannii*'s capacity to transfer this gene to other recipients through conjugation, with *Tn125* being the primary mechanism for its widespread distribution [72]. MBLs, such as *bla*_NDM-1_, *bla*_IMP_, and *bla*_SIM_, provide a significantly higher level of resistance to carbapenems, approximately 100–1000 times more resistance than OXA-type carbapenemases [5, 73]. Furthermore, *bla*_NDM-1_ imparts resistance not only to carbapenems but also to penicillins and cephalosporins [74]. Similarly, *bla*_OXA-23-like_ confers resistance to carbapenems, oxyiminocephalosporins, piperacillin, aminopenicillins, oxacillin, and aztreonam [5, 9, 16, 75]. This may provide a plausible explanation for the observed antibiotic resistance patterns, wherein cefoperazone/sulbactam, piperacillin/tazobactam, and cefepime exhibit almost 100% susceptibility in CS-ACB complex while being 100% resistant in CR isolates (Table 3) [69].

The presence of the *bla*_OXA-24-like_ gene was not detected in any of the isolates in this study. Although a recent study [76] reported the cooccurrence of *bla*_OXA-23-like_ and *bla*_OXA-24-like_ genes in a single isolate, most other studies did not observe such cooccurrence [21, 23, 66, 77]. The coexistence of *bla*_OXA-23-like_ and *bla*_NDM-1_ gene in our study might be the cause of the absence of the *bla*_OXA-24-like_ gene. Another possible explanation for the lack of the *bla*_OXA-24-like_ gene may be related to variations in sample size and the duration of the study. The earlier study that identified the presence of the *bla*_OXA-24-like_ gene covered a sampling period from 2012 to 2018, involving 382 *Acinetobacter* spp. [78]. In contrast, our study spanned only 6 months and included only 43 ACB complex isolates.

CR isolates presented a limited array of treatment options, with polymyxins (colistin and polymyxin B) being the sole available choices, despite concerns about their previously documented side effects [79]. In our study, both colistin and polymyxin B exhibited 100% susceptibility (Table 2), while tigecycline exhibited the next highest level of susceptibility at 60.47%. Like some of the past research [21, 23, 78], this investigation was unable to investigate clonal types through multilocus sequence typing (MLST) or whole genome sequencing because of insufficient funds, which is a major limitation of this study. However, three samples with intriguing results (AST and genes) underwent whole genome sequencing, and the results will be published separately as a case study. Additionally, it is important to note that this research did not provide details about patient outcomes following infection, primarily because most cases were successfully treated using third-line antibiotics. Lastly, due to inadequate funding, minimum inhibitory concentration (MIC) testing of carbapenem or other antibiotics could not be conducted.

This research emphasized the growing challenge posed by the CR-ACB complex in clinical environments. The CR-ACB complex, when isolated, demonstrated resistance to broad categories of antibiotics, indicating a significant issue [13]. Precise microbial diagnostic techniques are essential for the accurate identification of this organism to facilitate appropriate treatments [80]. Managing and minimizing infections caused by this organism can be effectively achieved through regular fumigation of ICUs and hospital wards, routine monitoring of organisms in these areas, and a judicious restriction on the use of antibiotics [81].

## 5. Conclusion

In summary, the objective of this study was to evaluate the presence of three genes associated with CR within the ACB complex at Kathmandu Model Hospital, Nepal. The results indicated a prevalence of 4.334% for the ACB complex. Respiratory samples, particularly from patients on mechanical ventilation in the ICUs, were the primary source of most ACB complex isolates. The study highlighted that the *bla*_OXA-23-like_ and *bla*_NDM-1_ genes are the most frequently occurring CR genes in Kathmandu Model Hospital, and there is an apparent increase in the prevalence of the *bla*_NDM-1_ gene. Furthermore, except for third-line antibiotics, many other antibiotics showed either no susceptibility or reduced susceptibility in the CR-ACB complex. This implies that the CR-ACB complex is going to be a formidable foe for clinicians. Therefore, it is recommended that all healthcare providers make a united effort through rigorous execution of infection prevention and control measures, prompt diagnosis, and responsible use of antibiotics to lessen the impact of AMR on both patients and healthcare facilities.

## Figures and Tables

**Table 1:** Gender-wise distribution of ACB complex among positive specimens.

**Type of specimens**	**Number of isolates**		**Total (no. [%])**
	**Male**	**Female**	
Tracheal aspirate	6	2	8 (18.6)
Sputum	6	2	8 (18.6)
ET tip	3	2	5 (11.63)
Pus	1	4	5 (11.63)
Blood	3	2	5 (11.63)
Wound swab	2	2	4 (9.302)
Tissue	2	1	3 (6.98)
CSF	1	0	1 (2.325)
Bile	0	1	1 (2.325)
CT tip	1	0	1 (2.325)
Urine	0	1	1 (2.325)
Bedsore	1	0	1 (2.325)
	26 (60.47%)	17 (39.53%)	43 (100%)

**Table 2:** Antibiotic susceptibility profile of ACB complex (*n* = 43) against 16 different antibiotics.

**Antibiotics**	**Antibiotic susceptible pattern (** **n** = 43**)**	
	**Susceptible isolate**	**Resistant isolate**
	**No. (%)**	**No. (%)**
Colistin (10 *μ*g)	43 (100)	0 (0)
Polymyxin B (300 units)	43 (100)	0 (0)
Tigecycline (15 *μ*g)	26 (60.47)	17 (39.53)
Amikacin (30 *μ*g)	15 (34.88)	28 (65.11)
Gentamicin (10 *μ*g)	13 (30.23)	30 (69.77)
Levofloxacin (5 *μ*g)	11 (25.58)	32 (74.42)
Cotrimoxazole (1.25/23.75 *μ*g)	9 (20.93)	34 (79.04)
Meropenem (10 *μ*g)	8 (18.6)	35 (81.4)
Imipenem (10 *μ*g)	8 (18.6)	35 (81.4)
Doxycycline (30 *μ*g)	8 (18.6)	35 (81.4)
Cefoperazone/sulbactam (100/10 *μ*g)	8 (18.6)	35 (81.4)
Ciprofloxacin (5 *μ*g)	8 (18.6)	35 (81.4)
Cefepime (30 *μ*g)	7 (16.28)	36 (83.72)
Piperacillin/tazobactam (75/30 *μ*g)	7 (16.28)	36 (83.72)
Ceftriaxone (30 *μ*g)	0 (0)	43 (100)
Cefotaxime (30 *μ*g)	0 (0)	43 (100)

**Table 3:** Difference in antimicrobial susceptibility pattern of CR-ACB and CS-ACB complex as well as chi-square test *p* values (*p* > 0.05) to assess the association between antimicrobial susceptibility of other antibiotics and carbapenem resistance.

**Antibiotics**	**CR-ACB complex (** **n** = 35**)**	**CS-ACB complex (** **n** = 8**)**	**p** ** value**
	**Resistivity (no. [%])**	**Resistivity (no. [%])**	
Tigecycline	17 (48.6)	0 (0)	0.014
Amikacin	28 (80)	0 (0)	< 0.001
Gentamicin	30 (85.7)	0 (0)	< 0.001
Levofloxacin	32 (91.4)	0 (0)	< 0.001
Cotrimoxazole	34 (97.1)	0 (0)	< 0.001
Doxycycline	32 (91.4)	3 (37.5)	0.003
Cefoperazone/sulbactam	34 (97.1)	1 (12.5)	< 0.001
Ciprofloxacin	34 (97.1)	1 (12.5)	< 0.001
Cefepime	34 (97.1)	1 (12.5)	< 0.001
Piperacillin/tazobactam	34 (97.1)	0 (0)	< 0.001
Colistin	0 (0)	0 (0)	< 0.001
Polymyxin B	0 (0)	0 (0)	< 0.001
Ceftriaxone	35 (100)	8 (100)	< 0.001
Cefotaxime	35 (100)	8 (100)	< 0.001

**Table 4:** Carbapenemase production in CR-ACB complex isolates and the presence of *bla*_NDM-1_ gene.

**Carbapenemase production**	**PCR result of *bla***_**NDM-1**_ **gene**		**Total (no. [%])**
	**Positive (no. [%])**	**Negative (no. [%])**	
Producers	12 (36.36)	21 (63.64)	33 (100)
Nonproducers	0 (0)	2 (100)	2 (100)

**Table 5:** Carbapenemase production in CR-ACB complex isolates and the presence of *bla*_OXA-23-like_ gene.

**Carbapenemase production**	**PCR result of *bla***_**OXA-23-like**_ **gene**		**Total (no. [%])**
	**Positive (no. [%])**	**Negative (no. [%])**	
Producers	29 (87.87)	4 (12.13)	33 (100)
Nonproducers	0 (0)	2 (100)	2 (100)

## Data Availability

Data of this research is available on NCBI under code Bioproject PRJNA951267.

## Supporting Information

Supporting Information Additional supporting information can be found online in the Supporting Information section. Table S1: primers used for PCR amplification. Table S2: PCR conditions for various genes. Table S3: specimen-wise distribution of carbapenem-resistant ACB complex.

## References

[1] Visca P., Seifert H., Towner K. J. (2011). Critical review *Acinetobacter* infection – an emerging threat to human health. *IUBMB Life*.

[2] Dhanoa A., Rajasekaram G., Lean S. S., Cheong Y. M., Thong K. L., Wong H. C. (2015). Endemicity of *Acinetobacter calcoaceticus-baumannii* complex in an intensive care unit in Malaysia. *Journal of Pathogens*.

[3] Hsieh W., Wang N. Y., Feng J. A., Weng L. C., Wu H. H. (2014). Types and prevalence of carbapenem-resistant *Acinetobacter calcoaceticus*-*Acinetobacter baumannii* complex in Northern Taiwan. *Antimicrobial Agents and Chemotherapy*.

[4] Ko K. S., Suh J. Y., Kwon K. T. (2007). High rates of resistance to colistin and polymyxin B in subgroups of *Acinetobacter baumannii* isolates from Korea. *The Journal of Antimicrobial Chemotherapy*.

[5] Peleg A. Y., Seifert H., Paterson D. L. (2008). *Acinetobacter baumannii*: emergence of a successful pathogen. *Clinical Microbiology Reviews*.

[6] Fishbain J., Peleg A. Y. (2010). Treatment of *Acinetobacter* infections. *Clinical Infectious Diseases*.

[7] Kanafani Z. A., Zahreddine N., Tayyar R. (2018). Multi-drug resistant *Acinetobacter* species: a seven-year experience from a tertiary care center in Lebanon. *Antimicrobial Resistance and Infection Control*.

[8] Asif M., Alvi I. A., Rehman S. U. (2018). Insight into *Acinetobacter baumannii*: pathogenesis, global resistance, mechanisms of resistance, treatment options, and alternative modalities. *Infection and Drug Resistance*.

[9] Ambler R. (1980). The structure of beta-lactamases. *Philosophical Transactions of the Royal Society B*.

[10] Bush K., Jacoby G. A., Medeiros A. A. (1995). A functional classification scheme for beta-lactamases and its correlation with molecular structure. *Antimicrobial Agents and Chemotherapy*.

[11] Bush K., Jacoby G. A. (2010). Updated functional classification of beta-lactamases. *Antimicrobial Agents and Chemotherapy*.

[12] Sawa T., Kooguchi K., Moriyama K. (2020). Molecular diversity of extended-spectrum *β*-lactamases and carbapenemases, and antimicrobial resistance. *Journal of Intensive Care*.

[13] Nguyen M., Joshi S. G. (2021). Carbapenem resistance in *Acinetobacter baumannii*, and their importance in hospital-acquired infections: a scientific review. *Journal of Applied Microbiology*.

[14] Ramadan R. A., Gebriel M. G., Kadry H. M., Mosallem A. (2018). Carbapenem-resistant *Acinetobacter baumannii* and *Pseudomonas aeruginosa*: characterization of carbapenemase genes and E-test evaluation of colistin-based combinations. *Infection and Drug Resistance*.

[15] Grisold A. J., Luxner J., Bedenić B. (2021). Diversity of oxacillinases and sequence types in carbapenem-resistant *Acinetobacter baumannii* from Austria. *International Journal of Environmental Research and Public Health*.

[16] Evans B., Amyes S. G. B. (2014). OXA *β*-lactamases. *Clinical Microbiology Reviews*.

[17] Shrestha S., Tada T., Shrestha B. (2015). Phenotypic characterization of multidrug-resistant *Acinetobacter baumannii* with special reference to metallo-*β*-lactamase production from the hospitalized patients in a tertiary care hospital in Nepal. *Journal of Institute of Medicine Nepal (JIOMN)*.

[18] Robledo I. E., Aquino E. E., Santé M. I. (2010). Detection of KPC in Acinetobacter spp. in Puerto Rico. *Antimicrobial Agents and Chemotherapy*.

[19] NIHR Global Health Research Unit on Genomic Surveillance of AMR (2020). Whole-genome sequencing as part of national and international surveillance programmes for antimicrobial resistance: a roadmap. *BMJ Global Health*.

[20] Young C. C. W., Karmacharya D., Bista M. (2022). Antibiotic resistance genes of public health importance in livestock and humans in an informal urban community in Nepal. *Scientific Reports*.

[21] Shrestha S., Tada T., Miyoshi-akiyama T. (2015). Molecular epidemiology of multidrug-resistant *Acinetobacter baumannii* isolates in a university hospital in Nepal reveals the emergence of a novel epidemic clonal lineage. *International Journal of Antimicrobial Agents*.

[22] Shrestha S., Tada T., Shrestha B. (2016). Emergence of aminoglycoside resistance due to armA methylase in multi-drug resistant *Acinetobacter baumannii* isolates in a university hospital in Nepal. *Journal of Nepal Health Research Council*.

[23] Joshi P. R., Acharya M., Kakshapati T., Leungtongkam U., Thummeepak R. (2017). Genes of *Acinetobacter baumannii* isolated from Nepal: antimicrobial resistance and clinical significance. *Antimicrob Resist Infect Control Control*.

[24] Shrestha M., Khanal B. (2013). Acinetobacter species: phenotypic characterization and antimicrobial resistance. *Journal of Nobel Medical College*.

[25] Thapa P., Bhandari D., Shrestha D. (2017). A hospital based surveillance of metallo-beta-lactamase producing gram negative bacteria in Nepal by imipenem-EDTA disk method. *BMC Research Notes*.

[26] Shrestha R. K., Dahal R. K., Mishra S. K. (2013). Ventilator associated pneumonia in tertiary care hospital, Mahargunj, Kathmandu, Nepal. *Journal of Institute of Medicine Nepal*.

[27] Mahto M., Chaudhary M., Shah A., Show K. L., Moses F. L., Stewart A. G. (2021). High antibiotic resistance and mortality with Acinetobacter species in a tertiary hospital, Nepal. *Public Health Action*.

[28] Sharma M., Sapkota J., Jha B., Mishra B., Bhatt C. P. (2019). Biofilm formation and extended-spectrum beta-lactamase producer among Acinetobacter species isolated in a tertiary care hospital: a descriptive cross-sectional study. *JNMA; Journal of the Nepal Medical Association*.

[29] Karn S., Pant N. D., Neupane S., Khatiwada S., Basnyat S., Shrestha B. (2017). Prevalence of carbapenem resistant bacterial strains isolated from different clinical samples: study from a tertiary care hospital in Kathmandu, Nepal. *Journal of Biomedical Science*.

[30] Shrestha D., Sherchand S. P., Gurung K., Manandhar S., Shrestha B., Sherchan S. P. (2016). Prevalence of multidrug resistant extended-spectrum ß-lactamase-producing bacteria from different clinical specimens in Kathmandu Model Hospital, Kathmandu, Nepal. *EC Microbiology*.

[31] Karunasagar A., Maiti B., Shekar M., Shalini Shenoy M., Karunasagar I. (2011). Prevalence of OXA-type carbapenemase genes and genetic heterogeneity in clinical isolates of Acinetobacter spp. from Mangalore, India. *Microbiology and Immunology*.

[32] Hu Q., Hu Z., Li J., Tian B., Xu H., Li J. (2011). Detection of OXA-type carbapenemases and integrons among carbapenem-resistant Acinetobactor baumannii in a teaching hospital in China. *Journal of Basic Microbiology*.

[33] Sahu C., Jain V., Mishra P., Prasad K. N. (2018). Clinical and laboratory standards institute versus European committee for antimicrobial susceptibility testing guidelines for interpretation of carbapenem antimicrobial susceptibility results for Escherichia coli in urinary tract infection (UTI). *Journal of Laboratory Physicians*.

[34] Clinical and Laboratory Standards Institute (CLSI) (2018). *M45-Methods for Antimicrobial Dilution and Disk Susceptibility Testing of Infrequently Isolated or Fastidious Bacteria. Vol. 35*.

[35] Piewngam P., Kiratisin P. (2014). Comparative assessment of antimicrobial susceptibility testing for tigecycline and colistin against *Acinetobacter baumannii* clinical isolates, including multidrug-resistant isolates. *International Journal of Antimicrobial Agents*.

[36] Kumar A. V., Pillai V. S., Dinesh K. R., Karim S. (2011). The phenotypic detection of carbapenemase in meropenem resistant Acinetobacter calcoaceticus–baumannii complex in a tertiary care hospital in South India. *Journal of Clinical and Diagnostic Research*.

[37] Minas K., Mcewan N. R., Newbold C. J., Scott K. P. (2011). Optimization of a high-throughput CTAB-based protocol for the extraction of qPCR-grade DNA from rumen fluid, plant and bacterial pure cultures. *FEMS Microbiology Letters*.

[38] Willner D., Daly J., Whiley D., Grimwood K., Wainwright C. E., Hugenholtz P. (2012). Comparison of DNA extraction methods for microbial community profiling with an application to pediatric bronchoalveolar lavage samples. *PLoS One*.

[39] Karthikeyan K., Thirunarayan M. A., Krishnan P. (2010). Coexistence of blaOXA-23 with blaNDM-1 and armA in clinical isolates of *Acinetobacter baumannii* from India. *The Journal of Antimicrobial Chemotherapy*.

[40] Klindworth A., Pruesse E., Schweer T. (2013). Evaluation of general 16S ribosomal RNA gene PCR primers for classical and next-generation sequencing-based diversity studies. *Nucleic Acids Research*.

[41] Ul-Hasan S., Bowers R. M., Figueroa-Montiel A. (2019). Community ecology across bacteria, archaea and microbial eukaryotes in the sediment and seawater of coastal Puerto Nuevo, Baja California. *PLoS One*.

[42] Bolyen E., Rideout J. R., Dillon M. R. (2019). Reproducible, interactive, scalable and extensible microbiome data science using QIIME 2. *Nature Biotechnology*.

[43] Quast C., Pruesse E., Yilmaz P. (2012). The SILVA ribosomal RNA gene database project: improved data processing and web-based tools. *Nucleic Acids Research*.

[44] Apic (2010). *Guide to the Elimination of Acinetobacter baumannii Transmission in Healthcare Settings*.

[45] Amatya R., Acharya D. (2015). Prevalence of tigecycline resistant multidrug resistant Acinetobacter calcoaceticus-*Acinetobacter baumannii* complex from a tertiary care hospital in Nepal. *Nepal Medical College Journal*.

[46] Parajuli N. P., Acharya S. P., Mishra S. K., Parajuli K., Rijal B. P., Pokhrel B. M. (2017). High burden of antimicrobial resistance among gram negative bacteria causing healthcare associated infections in a critical care unit of Nepal. *Antimicrobial Resistance and Infection Control*.

[47] Neupane L., Sah A. K., Rayamajhee B., Pokhrel A., Singh A. (2022). Detection of bla oxa-23 gene from carbapenem-resistant *Acinetobacter baumannii*. *Journal of Nepal Health Research Council*.

[48] Raut S., Rijal K. R., Khatiwada S. (2020). Trend and characteristics of *Acinetobacter baumannii* infections in patients attending Universal College of Medical Sciences, Bhairahawa, Western Nepal: a longitudinal study of 2018. *Infection and Drug Resistance*.

[49] Kumari M., Bhattarai N. R., Rai K., Pandit T. K., Khanal B. (2022). Multidrug-resistant Acinetobacter: detection of ESBL, MBL, blaNDM-1 genotype, and biofilm formation at a tertiary care hospital in Eastern Nepal. *International Journal of Microbiology*.

[50] Gupta N., Gandham N., Jadhav S., Mishra R. (2015). Isolation and identification of Acinetobacter species with special reference to antibiotic resistance. *Journal of Natural Science, Biology and Medicine*.

[51] Van T. D., Dinh Q. D., Vu P. D. (2014). Antibiotic susceptibility and molecular epidemiology of Acinetobacter calcoaceticus-baumannii complex strains isolated from a referral hospital in northern Vietnam. *Journal of Global Antimicrobial Resistance*.

[52] Nocera F. P., Attili A. R., De Martino L. (2021). *Acinetobacter baumannii*: its clinical significance in human and veterinary medicine. *Pathogens*.

[53] Rajkumari S., Pradhan S., Sharma D., Jha B. (2020). Prevalence and antibiogram of Acinetobacter species isolated from various clinical samples in a tertiary care hospital. *Journal of College of Medical Sciences*.

[54] Babapour E., Haddadi A., Mirnejad R., Angaji S. A., Amirmozafari N. (2016). Biofilm formation in clinical isolates of nosocomial *Acinetobacter baumannii* and its relationship with multidrug resistance. *Asian Pacific Journal of Tropical Biomedicine*.

[55] Sohail M., Rashid A., Aslam B. (2016). Antimicrobial susceptibility of acinetobacter clinical isolates and emerging antibiogram trends for nosocomial infection management. *Revista da Sociedade Brasileira de Medicina Tropical*.

[56] Hitsni R. N., Goldstein L. S., Arroliga A. C. (1999). Risk factors for an outbreak of multi-drug-resistant Acinetobacter nosocomial pneumonia among intubated patients. *Chest*.

[57] McDonald L. C., Banerjee S. N., Jarvis W. R., System N. N. I. S. (1999). Seasonal variation of Acinetobacter infections: 1987–1996. *Clinical Infectious Diseases*.

[58] Khanal B. R., Wagle S., Tiwari B. R. (2018). High susceptibility of Fosfomycin to Uropathogenic Escherichia coli Isolated at tertiary care hospital of Nepal. *National Journal of Laboratory Medicine*.

[59] Acosta J., Merino M., Viedma E. (2011). Multidrug-resistant *Acinetobacter baumannii* harboring OXA-24 carbapenemase, Spain. *Emerging Infectious Diseases*.

[60] Urban C., Go E., Mariano N., Rahal J. J. (1995). Interaction of sulbactam, clavulanic acid and tazobactam with penicillin-binding proteins of imipenem-resistant and -susceptible *Acinetobacter baumannii*. *FEMS Microbiology Letters*.

[61] Bhandari P., Thapa G., Pokhrel B. M., Bhatta D. R., Devkota U. (2015). Nosocomial isolates and their drug resistant pattern in ICU patients at National Institute of Neurological and Allied Sciences, Nepal. *International Journal of Medical Microbiology*.

[62] Ghimire U., Kandel R., Neupane M. (2021). Biofilm formation and blaOXA genes detection among *Acinetobacter baumannii* from clinical isolates in a Tertiary Care Kirtipur Hospital, Nepal. *Progress In Microbes & Molecular Biology*.

[63] Chaudhari B. K., Singh G. K., Parajuli K. P., Shrestha K. (2016). Incidence and susceptibility of uropathogens isolated among the patients at tertiary care hospital in Eastern Nepal. *Journal of Nobel Medical College*.

[64] Yuan Q., Xia P., Xiong L. (2023). First report of coexistence of blaKPC-2-, blaNDM-1- and mcr-9-carrying plasmids in a clinical carbapenem-resistant Enterobacter hormaechei isolate. *Frontiers in Microbiology*.

[65] Gao Y., Du P., Zhang P. (2023). Dynamic evolution and transmission of a blaNDM-1-bearing fusion plasmid in a clinical Escherichia coli. *Microbiological Research*.

[66] Villacís J. E., Bovera M., Romero-Alvarez D. (2019). NDM-1 carbapenemase in *Acinetobacter baumannii* sequence type 32 in Ecuador. *New Microbes New Infections*.

[67] Ramoul A., Loucif L., Bakour S., Amiri S., Dekhil M., Rolain J. M. (2016). Co-occurrence of blaNDM-1 with blaOXA-23 or blaOXA-58 in clinical multidrug-resistant *Acinetobacter baumannii* isolates in Algeria. *Journal of Global Antimicrobial Resistance*.

[68] Manandhar S., Zellweger R. M., Maharjan N. (2020). A high prevalence of multi-drug resistant gram-negative bacilli in a Nepali tertiary care hospital and associated widespread distribution of extended-spectrum beta-lactamase (ESBL) and carbapenemase-encoding genes. *Annals of Clinical Microbiology and Antimicrobials*.

[69] Martinez T., Martinez I., Vazquez G. J., Aquino E. E., Robledo I. E. (2016). Genetic environment of the KPC gene in *Acinetobacter baumannii* ST2 clone from Puerto Rico and genomic insights into its drug resistance. *Journal of Medical Microbiology*.

[70] Hakemi Vala M., Hallajzadeh M., Hashemi A. (2014). Detection of Ambler class A, B and D ß-lactamases among Pseudomonas aeruginosa and *Acinetobacter baumannii* clinical isolates from burn patients. *Annals of Burns and Fire Disasters*.

[71] Lee K., Chong Y., Shin H. B., Kim Y. A., Yong D., Yum J. H. (2001). Modified Hodge and EDTA-disk synergy tests to screen metallo-*β*-lactamase-producing strains of Pseudomonas and Acinetobacter species. *Clinical Microbiology and Infection*.

[72] Walsh T. R., Weeks J., Livermore D. M., Toleman M. A. (2011). Dissemination of NDM-1 positive bacteria in the New Delhi environment and its implications for human health: an environmental point prevalence study. *The Lancet Infectious Diseases*.

[73] Poirel L., Nordmann P. (2006). Carbapenem resistance in *Acinetobacter baumannii*: mechanisms and epidemiology. *Clinical Microbiology and Infection*.

[74] Zhang H., Hao Q. (2011). Crystal structure of NDM-1 reveals a common *β*-lactam hydrolysis mechanism. *The FASEB Journal*.

[75] Paton R., Miles R. S., Hood J., Amyes S. G. B., Miles R. S., Amyes S. G. B. (1993). ARI 1: *β*-lactamase-mediated imipenem resistance in *Acinetobacter baumannii*. *International Journal of Antimicrobial Agents*.

[76] Hadjadj L., Bakour S., Rolain J. M. (2018). Co-occurrence of carbapenemase encoding genes in *Acinetobacter baumannii*, a dream or reality?. *BMC Microbiology*.

[77] Pillonetto M., Arend L., Vespero E. C. (2014). First report of NDM-1-producing *Acinetobacter baumannii* sequence type 25 in Brazil. *Antimicrobial Agents and Chemotherapy*.

[78] Manandhar S., Nguyen Q., Nguyen Thi Nguyen T. (2022). Genomic epidemiology, antimicrobial resistance and virulence factors of *Enterobacter cloacae* complex causing potential community-onset bloodstream infections in a tertiary care hospital of Nepal. *JAC-Antimicrobial Resistance*.

[79] Sampson T. R., Liu X., Schroeder M. R., Kraft C. S., Burd E. M., Weiss D. S. (2012). Rapid killing of *Acinetobacter baumannii* by polymyxins is mediated by a hydroxyl radical death pathway. *Antimicrobial Agents and Chemotherapy*.

[80] Meron G., Rae H., Shubha Y., Bradford C., Crane N. J. (2017). Accurate and rapid differentiation of *Acinetobacter baumannii* strains by Raman spectroscopy: a comparative study. *Journal of Clinical Microbiology*.

[81] Weinberg S. E., Villedieu A., Bagdasarian N., Karah N., Teare L., Elamin W. F. (2020). Control and management of multidrug resistant *Acinetobacter baumannii*: a review of the evidence and proposal of novel approaches. *Infection Prevention in Practice*.

