# Supplementary material for: Carbapenem Resistance in Acinetobacter calcoaceticus-baumannii Complex Isolates From Kathmandu Model Hospital, Nepal, Is Attributed to the Presence of blaOXA-23-like and blaNDM-1 Genes
Source: Biomed Res Int. 2024 Aug 12;2024:8842625. doi: 10.1155/2024/8842625 (PMC11333142; doi:10.1155/2024/8842625)
Supplement: Supporting Information — Additional supporting information can be found online in the Supporting Information section. Table S1: primers used for PCR amplification. Table S2: PCR conditions for various genes. Table S3: specimen-wise distribution of carbapenem-resistant ACB complex. [file 8842625.f1.docx]

**Supplementary Tables**

**Supplementary Table 1: Primers used for PCR amplification.**

| **Primer** | **Sequence** | **Reference** |
| --- | --- | --- |
| NDM-1 Forward | 5’- GGTTTGGCGATCTGGTTTTC -3’ | 28 |
| NDM-1 Reverse | 5’- CGAATGGCTCATCACGATC - 3' | 28 |
| Oxa-23-like Forward | 5’- GATCGGATTGGAGAACCAGA -3’ | 27 |
| Oxa-23-like Reverse | 5’- ACGGCTACCTTGTTACGACTT -3’ | 27 |
| Oxa-24-like Forward | 5’- GGTTAGTTGGCCCCCTTAA -3’ | 27 |
| Oxa-24-like Reverse | 5’- AGTTGAGCGAAAAGGGGATT -3’ | 27 |
| 16s rRNA with adapter sequence Forward | 5'-TCGTCGGCAGCGTCAGATGTGTATAAGAGACAGCCTACGGGNGGCWGCAG-3’ | 29 |
| 16s rRNA with adapter sequence Reverse | 5'-GTCTCGTGGGCTCGGAGATGTGTATAAGAGACAGGACTACHVGGGTATCTAATCC-3’ | 29 |

**Supplementary Table 2: PCR conditions for various genes**

| **Steps** | **Temperature** | **Duration** | **Cycle** |
| --- | --- | --- | --- |
| **NDM-1** | | | |
| Initial Denaturation | 95⁰C | 2 minutes | 1 |
| Denature | 95⁰C | 30 secs | 30 |
| Annealing | 56.3⁰C | 30 secs |  |
| Extension | 72⁰C | 70 secs |  |
| Final extension | 72⁰C | 5 minutes | 1 |
|  |  |  |  |
| **OXA-23-like** | | | |
| Initial Denaturation | 95⁰C | 2 minutes | 1 |
| Denature | 95⁰C | 30 secs | 14 |
| Annealing | 59.2⁰C | 30 secs |  |
| Extension | 72⁰C | 60 secs |  |
| Denature | 95⁰C | 30 secs | 19 |
| Annealing | 52.2⁰C | 30 secs |  |
| Extension | 72⁰C | 60 secs |  |
| Final extension | 72⁰C | 5 minutes | 1 |
|  |  |  |  |
| **OXA-24-like** | | | |
| Initial Denaturation | 95⁰C | 2 minutes | 1 |
| Denature | 95⁰C | 30 secs | 30 |
| Annealing | 54.2⁰C | 30 secs |  |
| Extension | 72⁰C | 30 secs |  |
| Final extension | 72⁰C | 5 minutes | 1 |
|  |  |  |  |
| **16s rRNA** | | | |
| Initial Denaturation | 95⁰C | 2 minutes | 1 |
| Denature | 95⁰C | 30 secs | 30 |
| Annealing | 55⁰C | 30 secs |  |
| Extension | 72⁰C | 30 secs |  |
| Final extension | 72⁰C | 5 minutes | 1 |

**Supplementary Table 3: Specimen wise distribution of carbapenem resistant ACB complex.**

| **Specimens** | **Carbapenem resistant isolate** | **Carbapenem susceptible isolate** |
| --- | --- | --- |
|  | **No. (%)** | **No. (%)** |
| Sputum | 7 (87.5) | 1 (12.5) |
| Tracheal aspirate | 7 (87.5) | 1 (12.5) |
| Pus | 5 (100) | 0 (0) |
| ET tip | 4 (80) | 1 (20) |
| Blood | 3 (60) | 2 (40) |
| Tissue | 3 (100) | 0 (0) |
| Wound swab | 2 (50) | 2 (50) |
| Bed sore | 1 (100) | 0 (0) |
| Bile | 1 (100) | 0 (0) |
| CT tip | 1 (100) | 0 (0) |
| CSF | 1 (100) | 0 (0) |
| Urine | 0 (0) | 1 (100) |
| Total | 35 (81.4) | 8 (18.6) |
